# Review of Peak Detection Algorithms in Liquid-Chromatography-Mass Spectrometry

**DOI:** 10.2174/138920209789177638

**Published:** 2009-09

**Authors:** Jianqiu Zhang, Elias Gonzalez, Travis Hestilow, William Haskins, Yufei Huang

**Affiliations:** 1 Dept. of ECE, University of Texas at San Antonio, San Antonio, TX 78249, USA; 2 Dept. of Biology, University of Texas at San Antonio, San Antonio, TX 78249, USA; 3 Children's Cancer Research Institute, UT Health Science Center, San Antonio, TX 78229, USA

## Abstract

In this review, we will discuss peak detection in Liquid-Chromatography-Mass Spectrometry (LC/MS) from a signal processing perspective. A brief introduction to LC/MS is followed by a description of the major processing steps in LC/MS. Specifically, the problem of peak detection is formulated and various peak detection algorithms are described and compared.

## 1. INTRODUCTION

The identification and quantification of proteins in biological samples play a crucial role in biological and biomedical research [1-4]. For example, in biomarker discovery studies, the aim is to elucidate a set of proteins that can be used to reliably differentiate diseased and normal samples. Accurate protein identification and quantification are required to achieve this goal.

### 1.1. Liquid Chromatography/Mass Spectrometry (LC/MS) for Protein Identification and Quantification

The most powerful method for protein identification and quantification is Liquid Chromatography/Mass Spectrometry (LC/MS), a combination of Liquid Chromatography (LC) and Mass Spectrometry (MS) which are explained separately below.

**Fig. (1):**
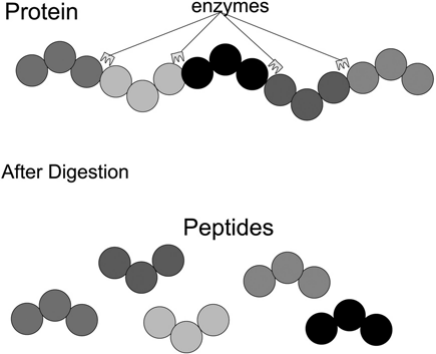
Proteins are digested into peptides.

After purification and separation, proteins can be cut into peptides by enzymes (see Fig. **1**) at theoretically predictable positions. Each protein generates a unique combination of peptides with different masses. Thus, by knowing the mass list of a protein, or “protein mass fingerprint”, a specific protein can be identified. **Mass Spectrometry** achieves protein identification and quantification through measuring the mass and abundance of peptides contained in a sample. There exist several databases (such as Swiss-Prot http://www.expasy.org/sprot/) that store the mass fingerprints of known proteins. Thus the protein content of a sample can be obtained by submitting a mass list to these databases.

However, MS has limitations in protein identification since different peptides may share the same mass. This, coupled with limited protein sequence coverage (the number of peptides detected by MS per protein), mass fingerprint matching can not uniquely resolve the identity of proteins. In such cases, MS/MS technologies can be employed. In MS/MS, peptides are further fragmented into smaller molecules. Similar to DNA sequencing, the fragmented molecules can be pieced back together to identify the exact amino acid sequence of a parent peptide.

**Fig. (2):**
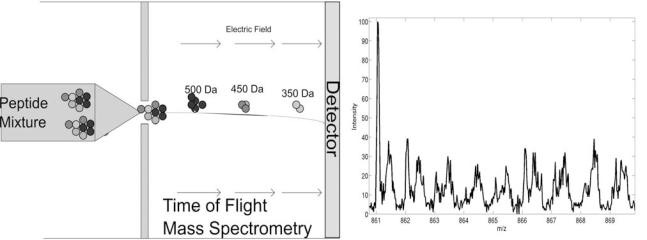
Time-of-Flight mass spectrometry.

There are several types of MS technologies for measuring peptide masses including: Fourier transform (FTMS) [5] and Time-of-Flight (TOFMS) [6]. In both cases, peptides are charged (ionized) through either Electrospray Ionization (ESI) [7] or Matrix-Assisted Laser Desorptation/Ionization (MALDI) [7, 8]. (Note that peptides from the same species may carry different number of charges (z). Multiple factors may affect the charge state distribution of a peptide which have not been well characterized). In FTMS, charged peptide ions are trapped in a magnetic field, where they are excited to a larger cyclotron radius by an oscillating electric field perpendicular to the magnetic field. The excitation results in the ions moving in a circle with a frequency determined by their mass/charge ratio (m/z) value. The resulting signal consists of the superposition of sine waves. Then the mass spectrum is extracted from this signal by performing a Fourier transform. In TOFMS (Fig. **2**), ions are accelerated by an electrical field to the same kinetic energy with the velocity of the ion depending on the m/z value. Thus time-of-flight can be used to translated to m/z. After estimating the number of charges (charge state estimation), the mass of the peptides can be extracted from the m/z values recorded in the mass spectrum which can be viewed as the superposition of mass finger prints of all proteins contained in the sample. In both MS technologies, the intensity of the recorded signal indicates the abundance of the peptides, which enables protein quantification. FTMS has a very high mass resolution, but due to cost and sensitivity issues, TOFMS is also very common.

In MS, the problem of ion-ion suppression is a serious problem in complex biological samples. In the ionization process, peptides compete for electric charges such that some low abundance peptides may not be ionized; consequently, they can not be identified and quantified. For this reason, peptide separation using Liquid Chromatography (LC) is employed to reduce the total amount of peptides entering the mass spectrometer at a give time. The combination of LC and MS (LC/MS) is rapidly emerging as a method of choice for large-scale biomarker discovery [9, 10].

**LC **is a laboratory technique for the separation of peptide mixtures by passing peptide samples through a column that contains a certain solvent. Depending on their physicochemical properties and interactions with the solvent, peptides travel through the column with different speeds. The elution (retention) time is the characteristic time it takes for a particular peptide to pass through the system (from the column inlet to the mass spectrometer) under set conditions. An illustration of the LC elution process is shown in Fig. (**3**).

In LC/MS, peptides are less likely to compete for charge since they are separated in elution time. Therefore, relative to MS, many more peptides can be measured. Sequence coverage (i.e. the number of peptides observed from a given protein) affects the confidence of protein identification and quantification. LC can also be combined with MS/MS to form LC/MS/MS. Note that MS instruments are often set up to collect LC/MS and LC/MS/MS data simultaneously.

### 1.2. Typical Workflow Using LC/MS

There are several common types of proteomic studies that may utilize LC/MS. After peak detection, comparative studies aim at selecting features (peptide signals) consistently correlated with a particular physiological status, such as development of disease [11]. The process of selecting differentially expressed peptide signals is called **feature selection**. Comparative study ends when features are selected. Second, targeted studies aim at the determination of peptide/protein identity and biological significance [12, 13]. Third, survey proteomic studies aim at the identification and quantification of all proteins contained in a biological sample [14, 15].

A biomarker discovery study can first employ comparative study for identifying features that can reliably differentiate one class of samples from another. Then a targeted study can be conducted to clarify the protein identity of selected features. A hypothesis driven study, on the other hand, can start with targeted study directly. A survey proteomic study can serve the purpose of creating a proteomic database, containing protein identities and contaminants, or as a first step before comparative study.

A typical workflow using LC/MS for biomarker discovery is depicted in Fig. (**4**). Protein extracts from different samples are digested by a protease (typically trypsin) to prepare samples of complex peptide mixtures. An aliquot of each sample is injected into the LC/MS system. The remainder of the sample is used for replicate injections and for future (targeted) LC/MS and LC/MS/MS analysis. After peak detection and alignment is performed by the LC/MS analysis software, a comparison between samples will enable the selection of those peaks that display differential behavior between samples. As data are collected in LC/MS mode only up to this point, the identity (i.e., the amino acid sequence) of the selected peaks (peptides) is yet unknown. For this, another aliquot of the sample is often injected onto a different LC/MS/MS system, where a tandem mass spectrometer collects MS/MS spectra from (differentially) expressed peptides. In this two-step, and often two-instrument, approach to biomarker discovery, quantitative and qualitative (sequence) information are collected separately by LC/MS and LC/MS/MS.

Although it is possible to discover biomarkers by LC/MS/MS directly it has several drawbacks. Since MS/MS scans are slower than MS scans, undersampling often occurs [16]. Also, the reproducibility of LC/MS/MS is poor. Overall, the limitations of 'shotgun proteomics' methods preclude replicate analysis required to gather statistical information from a large number of samples; therefore, LC/MS is the method of choice in many biological research problems.

### 1.3. Data Structure of LC/MS

Fig. (**5**) depicts an example of an LC/MS dataset from one LC/MS data run. In LC/MS, the output of the LC column is inducted to a mass spectrometer periodically throughout the elution process. The time points can be referred to as elution time sampling points $t\in \left\{t_{1},...,t_{T}\right\}$ if there are a total of *T* sampling time points. At each time sample point, the mass spectrometer will produce an MS scan which registers the m/z values and the corresponding abundance (intensity) of all ionized peptides. The *i* th scan can be represented as

$S_{t_{i}}=\left\{I_{t_{i},{\mathrm{mz}}_{1}},...,I_{t_{i},{\mathrm{mz}}_{z}},...,I_{t_{i},{\mathrm{mz}}_{Z}}\right\},$

where *Z* is the total number of detected m/z values and $z\in \left\{1,...,Z\right\}$ represents the index of the set of m/z values $\left\{{\mathrm{mz}}_{1},{\mathrm{mz}}_{2}...{\mathrm{mz}}_{Z}\right\}.$ $I_{t_{i},{\mathrm{mz}}_{z}}$ stands for the intensity of the spectrum at the *zth* m/z value *mz_z_* and elution time *t_i_*. Finally, $I=\left\{S_{t_{1}},...,S_{t_{T}}\right\}$ represents the complete set of scans of a particular LC/MS run. It is also possible to organize *I* as a collection of elution time profiles or Extracted Ion Chromatographs (XICs), i.e., $I=\left\{E_{{\mathrm{mz}}_{1}},...,E_{{\mathrm{mz}}_{z}},...,E_{{\mathrm{mz}}_{Z}}\right\},$

where $E_{{\mathrm{mz}}_{z}}=\left\{I_{t_{1},{\mathrm{mz}}_{z}},...,I_{t_{T},{\mathrm{mz}}_{z}}\right\}$ stands for the elution time profile or XIC at m/z value *mz_z_*.

Note that the sampling rate in the m/z dimension may vary from scan to scan and the length of MS scans may vary. The sampling m/z values also may vary from scan to scan. Thus, elution time profiles cannot be extracted directly. There are several ways of extracting the elution time profiles and we will discuss them in detail when we introduce each LC/MS algorithm. Since different peptide species are eluted out at different time intervals, they arrive at the mass spectrometer within different time periods and will form distinct chromatographic peaks in elution time profiles indexed by the m/z values *mz_z_*. Fig. (**6**) shows an example of a chromatographic peak in an elution time profile.

### 1.4. Signals Generated by a Peptide

A peptide species with molecular weight *m* may generate a group of related peaks in the LC/MS dataset. First, when a peptide species enter the mass spectrometer, different numbers of charges will be attached to them during the ionization process, which results in different charge states. If *H*^+^ is the weight of the charge at charge state *z* , the resulting m/z value can be calculated as $\mathrm{mz}=\left(m+z\times H^{+}\right)/z$ for $z\in \left\{1,2,...\right\}$. In ESI, the charge state can be higher than 30. In MALDI, the charge state is mostly one or two. The charge state distribution is determined by a variety of factors such as molecular weight, physicochemical properties of peptides as well as the instrument. Currently, it is generally not clear what charge state distribution will result when a particular peptide enters a mass spectrometer. Due to charge state dispersion, one peptide species may register peaks at a series of m/z locations. Sometimes, one charge state dominates, but it is also very common that two or more charge states occur with comparable peptide counts.

Apart from peptide charge state dispersion, each peptide species should register as a series of isotope peaks in MS. This is due to the fact that different chemical elements that form peptides have isotopes in the natural world. For example, while carbon C^12^ usually has 6 protons and 6 neutrons, it has an isotope with 6 protons and 7 neutrons (C^13^). The presence of C^13^ will increase the molecular weight of the corresponding peptide species to $m+w_{c}\times p$, where *w_c_* stands for the weight of the extra neutron and *p* is the number of C^13^s in the peptide. Given a total number of peptide counts of a peptide species, the percentage of the peptide composed of *p* carbon isotopes is governed by the Poisson distribution [17, 18], and is called an isotope pattern. It should be noted that other chemical elements (such as oxygen) may also contribute to the isotope pattern. However, C^13^ is the dominating factor in the formation of an isotope pattern.

There exist various approaches [19-21] addressing the calculation of isotope patterns. One of the most popular is based on “averagine”, an averaged molecular formula for peptides [19]. Using the “averagine” molecular formula, one can estimate the number of carbons, oxygens etc. contained in a peptide sequence given the total molecular mass, which in turn will allow for the calculation of an estimated isotope pattern. The presence of an isotope pattern predicted by the “averagine” is important evidence of the existence of a peptide since non-peptides that do not have similar chemical composition as the “averagine” will not have the same isotope pattern as that of peptides. In Fig. (**7**), we plot an example of an observed isotope pattern.

Isotope and charge state dispersion result in a phenomenon where multiple peaks will be registered for one peptide species in MS spectrums at different m/z locations. Also, at these m/z locations, similar chromatographic peaks will occur in their elution time profiles. These facts enormously complicate the accurate identification of peptide identity. However, before the peptide identity can be inferred based on isotopic pattern and charge state distributions, it is important to discern peaks that were generated by real peptides from those by random electrical and chemical noise.

LC/MS peaks occupy both the LC and MS dimensions, and a number of factors affect the peak shape. The MS peak shape is mainly determined by the mass spectrometer used and can be modeled as Gaussian although other more complicated models [22] provide a better fit. The MS peak width can be predicted by the resolution of the mass spectrometer which is described by the Full-Width-Half-Maximum (FWHM) ratio. For example, if an instrument has a resolution of 10,000 FWHM resolution, it means that at m/z value 2000 Dalton, the width of the peak at half of the maximum intensity can be calculated as 2000/10,000 Dalton. There is generally a linear relationship between the m/z value and MS peak width. The observed peak width will differ from the width predicted by the instrument resolution due to various reasons such as temperature and calibration [22].

While the MS peak shape is largely determined by the MS instrument, the LC peak shape is determined by more complex factors that have not been completely characterized. Some factors include the concentration of solvent or gradient used for chromatogram separation and physicochemical interactions between peptides. While some researchers consider the LC peaks as Gaussian shaped, our observation of LC/MS datasets suggests that LC peaks are bell-shaped like peaks with a long tail in a significant number of cases. On the other hand, we have also observed many other types of LC peak shapes, some of which have double local maxima as shown in Fig. (**8**).

**Table:** 

List of Terminologies	
MS	Mass Spectrometry of peptides or intact proteins
MS/MS	Mass Spectrometry of fragmented peptides
LC	Liquid-Chromatography, a method for separating peptides
FWHM	Full-Width-Half-Maximum, describes the resolution of MS
ESI	Electrospray Ionization, a method for ionizing peptide
MALDI	Matrix-Assisted Laser Desorptation/Ionization
FTMS	Fourier-Transform Mass Spectrometry
TOFMS	Time-of-Flight Mass Spectrometry
Feature	Peptide signals that can be used to classify samples
Peak Detection	The process of distinguishing peptide and noise peaks
Isotope Pattern	Abundant ratios of a peptide spices in different isotopes forms

## 2. PEAK DETECTION IN LC/MS

As the initial step in LC/MS data processing, peak detection aims to tease apart peaks generated by real peptides from those generated by random electrical and chemical noise. After peak detection, LC/MS datasets from different classes can be compared to extract features for classifying samples.

There are two essential aspects to consider when designing a peak detection algorithm. The first is to select a set of characteristics that can be utilized to differentiate peptide peaks and noise peaks. The second is to design a method to extract these characteristics from unknown peaks and compare them with that of known peptide peaks. We can categorize different peak detection algorithms based on the difference in these two aspects. One can also predict the performance of a peak detection algorithm based on these two aspects. Theoretically, if an algorithm explores all differentiating characteristics between peptide and noise peaks, and extracts these characteristics accurately, the peak detection performance will be the best. On the other hand, if an algorithm only utilizes one or two such characteristics, and if the extraction process is not done accurately, the performance is going to suffer. Listed below are the characteristics that have been employed to differentiate peptides from random noise.

- MS and LC Peak Intensity: Usually noise peaks are of low intensity and high abundance peptides peaks have intensities that are well beyond the noise level. However, it is generally impossible to differentiate low abundance peptide peaks and chemical noise peaks based on intensity only.
- MS Isotope Pattern: Peptides have predictable isotope patterns in the MS spectrum but noise peaks do not. This is a popular choice for many peak detection algorithms. However, for weak peptide peaks, only incomplete isotope patterns can be observed in the spectrum.
- Frequency Content of the MS Spectrum: Noise peaks, especially electrical noise, occupy the higher end of the frequency spectrum. Thus, by performing filtering, noise peaks and peptide peaks can be separated to a degree. However, issues arise when the resolution of the MS instrument is high since both peptide peaks and noise peaks occupy similar ranges of the spectrum and thus weak peptide peaks could also be filtered out.
- Frequency Content of the Elution Time Profile: The elution time profiles contain high frequency noise such as Poisson noise due to the discrete nature of the ion signal [23] or high frequency thermal noise. This type of noise is usually easy to differentiate from the low frequency LC peaks and can be removed by filtering methods.
- Length of LC Peaks: Since it takes time for all molecules of a peptide species to elute from the LC column, an LC peak usually lasts for a relatively long period of time. If an LC peak is too narrow, it is very likely that the peak is due random noise. However, there does not exist a comprehensive study on how to predict the length of LC peaks under various experimental conditions and it is generally a guess as to how to choose the cut-off length of peptide peaks. For a low abundance peptide, the elution time is usually shorter and it is hard to differentiate it from noise.
- Shape of LC Peaks: Some researchers [24, 25] consider LC peaks that match with a particular shape as peptide peaks. However, currently the shape of LC peaks is not predictable and varies greatly from one peptide to another. Peak detection based on the shape of LC peak will have low sensitivity.

Usually a peak detection algorithm combines one or more of the above characteristics to select peptide peaks. Obviously the choice of the subset of characteristics will affect the sensitivity and specificity of the algorithm greatly. For example, if the shape of LC peak is chosen, then the sensitivity of the algorithm will be greatly reduced since any LC peaks that do not conform to the predefined LC peak shape will be considered as non-peptide peaks. When peak intensity is chosen as the sole criterion, a low threshold on intensity will be employed if high sensitivity is desired which will greatly reduce the specificity of the algorithm.

The second aspect of a peak detection algorithm, i.e., the method for extracting the characteristics, will also affect the performance greatly. For example, for isotope pattern matching, one can chose to use matched filtering methods [26], or the maximum entropy methods [27] to determine the match between an expected and an observed isotope pattern.

In what follows, we will survey and compare popular peak detection algorithms in the literature based on these two aspects. We categorize algorithms based on their main processing technique. Most of these algorithms are distributed in open source software packages. Commercial algorithms are not included in this review for lack of publicly available details of their implementation.

### 2.1. Peak Detection Algorithms Based on Isotope Pattern

There exist a number of LC/MS peak detection and feature selection software packages that detect peaks mainly based on isotope pattern matching in the m/z dimension. These methods are also called 1-D LC/MS processing. Well-known software packages include VIPER [28], SuperHirn [29], OpenMS [30] and PepList [31]. Note that the peak detection algorithm in VIPER and SuperHirn is very similar and we only describe VIPER in this paper. A common characteristic of these algorithms is that they process MS scans first using peptide peak detection based on isotope pattern matching. We can view these methods as direct extensions to previous mass spectrometry peak picking algorithms [21]. Peak picking in the LC dimension is limited to simple noise filtering or thresholding. The main drawback of these 1-D approaches is that they do not utilize the 2-D nature of the LC/MS dataset. 1-D MS scans are usually noisy and peak picking based on isotope pattern matching may miss a lot of peptide peaks.

#### 2.1.1. Visual Inspection of Peak/Elution Relationships (VIPER)

VIPER is an algorithm package developed by the group at Pacific Northwest National Laboratory to perform peptide identification based on the accurate mass and time (AMT) tag approach [28]. In this approach, a database tagging the molecular mass and LC elution time of previously identified peptides is employed. VIPER is applied to the 2-D LC-MS data to extract LC-MS features in terms of mass and elution time and compare with the database of tagged peptides for protein identification.

The VIPER software package utilizes software such as ICR2LS that uses the THRASH algorithm for peak picking. The THRASH algorithm [21] differentiates peptides and noise peaks based on isotope pattern matching. It compares the expected isotope pattern to the observed isotope pattern in each MS scan to detect peptide peaks. A byproduct, called the isotopic fit score, will be reported for each detected isotope group. The score is based on the mean square error difference between an isotope pattern template and the observed data to signify the confidence of each detected peptide. It implicitly assumes a Gaussian noise model in the MS spectrums.

The isotope matching algorithm also sums up a group of isotope peaks and then maps the masses and intensities of the isotopes to those of monoisotopes, which effectively deisotopes each MS spectrum. Note that the intensity value of the deisotoped spectrum is the combined intensities of potential masses from all possible charge states.

No additional peak picking steps are employed in the LC dimension. The LC dimension processing is aimed at reporting the mass and elution time tag of each detected peptide species. VIPER assumes that peaks from the same peptide species are similar in both the m/z and LC time dimensions. Consequently, it performs a clustering step to group the peaks of the same species together. To this end, a single link hierarchical clustering is applied with the distance (dissimilarity) measure defined on the features (mass, intensity, elution time, and isotopic fit score) and expressed as

(1) $$d^{2}\left(a,b\right)=w_{\mathrm{mass}}{\left(m_{a}-m_{b}\right)}^{2}+w_{\mathrm{int}}{\left(I_{a}-I_{b}\right)}^{2}+w_{\mathrm{ET}}{\left(t_{a}-t_{b}\right)}^{2}+w_{\mathrm{fit}}{\left(f_{a}-f_{b}\right)}^{2}$$

where* a* and *b* are two points on the 2-D spectra with *m*, *I*, *t*, and *f* being the respective features, and *w_mass_*, *w_int_*, *w_ET_*, and *w _fit_* are built-in weights assigned to reflect the importance of each feature in contributing to the detection of peptide peaks. After clustering, a single set of cluster features is created for each cluster, which contains the median mass, the central normalized elution time (NET), and an intensity estimate. The central elution time is based on the scan number containing the highest peak intensity and the intensity feature is the estimated as the area under the LC/MS peak intensity over the time scan of the cluster. VIPER actually provides two options for calculating the cluster feature: it can be based on data either from combined charged states or the charge state with the highest overall intensity and the latter is suggested by VIPER. These cluster features are then compared with the selected AMT tag databases for peptide/protein identification.

#### 2.1.2. PepList

Peplist [31] is the peak detection algorithm in the LC/MS data processing software package SpecArray. The algorithm differentiates peptide and non-peptide peaks based on isotope matching, intensity of MS peaks, intensity of LC peaks, frequency content of elution, and MS peaks. A one-dimensional translation-invariant wavelet transformation filtering method (Symmlet8) [32] is used to remove high frequency noise in the MS scans. A smoothing method developed in [33] is used to smooth elution time profiles. Local background noise level is estimated as the median intensity within a window of 50 m/z. Any MS peak with a signal to noise ratio of less than 2 will be considered as noise and removed. The algorithm further assumes that peptide features form isotope patterns in the MS scans, and the most intense peak could be the monoisotopic *M* + 1 or *M* + 2 isotopic peak in an isotope pattern. Isotope pattern matching based on these assumptions is used to pick peptide features. The algorithm further assumes that the highest peak in an isotope pattern must have an SNR of greater than 5. The algorithm assumes that a peptide feature must have LC peaks with an SNR of greater than 2 when measured at the apex of the LC peak, and any LC peaks with smaller SNR are considered as noise.

After the algorithm performs wavelet filtering for each scan in the LC/MS dataset, a centroid MS spectrum is generated for each scan by locating all local maxima. Next, local background noise is estimated using a window size of 50 m/z and stored. With estimated noise level, the SNR threshold of 2 is applied to the centroid peak list in each scan. Next, all peaks in each scan are ordered by intensity. Starting from the highest peak, the algorithm first estimates the charge state of the peak by checking the m/z difference of the considered peak and its neighboring MS peaks. Then each peak considered is assumed to be the monoisotopic *M* + 1 or *M* + 2 isotopic peak and isotope matching is performed sequentially. Once a good match is found, a peptide feature will be reported. The criteria for a “good” match is not described and it is also not clear if all peaks belonging to the identified isotope pattern will be removed from the list of peaks. Afterwards, the algorithm proceeds to process the next highest peak in the scan having an SNR of at least 5. The process is repeated for all scans.

For each identified peptide feature in the MS scans, a single ion chromatogram (SIC) is constructed by summing the first 3 isotopic peaks and tracing the sum intensity along the LC dimension. A smoothing procedure developed in [33] is used to remove noise, and the noise level is estimated subsequently. LC peaks with SNR less than 2 will be considered as non-peptide or noise peaks. Finally the algorithm reports the area of the SIC as the abundance of the peptide and the apex location as the retention time.

### 2.2. Peak Detection Algorithms Based on Peak Shape

These algorithms make assumptions on either the LC peak or the 2-D peptide peak shape. Based on the shape assumptions, these algorithms first filter out peaks that do not conform to these assumptions as noise peaks. After this filtering step, the algorithms may utilize other characteristics such as intensity and isotope pattern matching to further reduce the candidate peptide list. The main drawback of this type of algorithm is that the real peptide peak shape in the LC dimension is hard to predict and LC peaks that do not conform to the shape assumption will be missed by these algorithms.

#### 2.2.1. Matched Filtration with Experimental Noise Determination (MEND)

The MEND peak picking algorithm [25] attempts to develop a denoising and peak picking filter that enables low-intensity and low-S/N peaks to be accurately determined.

The algorithm assumes the shape of the chromatographic peak to be Gaussian, and it differentiates peptide and noise peaks in the elution time profiles (XICs) based on matched filtering. Besides LC peak shape, the algorithm also assumes that the maximum point of a peptide's LC peak must intersect the maximum point of a MS peak. Lastly, the algorithm will further differentiate peptide and non-peptide peaks based on isotope pattern fitting. The algorithm will examine these three characteristics for each peak candidate and report a fitting score for each of the characteristics. Finally a summarizing score will be generated and a threshold is applied to differentiate peptide and noise.

To perform matched filtering, blank XICs without any LC peaks are used for estimating noise power spectral density *P_NN_*(*f*). Then matched filtering is applied using the transfer function *H*(*f*)=S^*^(*f*)/*P_NN_*(*f*), where S^*^(*f*) is the conjugated Fourier transform of a Gaussian shaped curve. Each XIC *I_mz_* $\forall \mathrm{mz}\in \left\{{\mathrm{mz}}_{1},...,{\mathrm{mz}}_{Z}\right\}$ will be filtered using the transfer function and the output is the filtered XICs. After matched filtering, a fixed number of LC peaks in each XIC are considered as peptide candidates and each peak candidate is assigned with a fitting score *S_c_* =(*S/N*)*G*, where (*S/N*) is the estimated signal to noise ratio and $G=0.67\sqrt{n}$ is the gain due to matched filtering and *n* is the number of data points per chromatographic peak.

The algorithm assumes that the maximum point of an LC peak must intersect the maximum point of an MS peak. A score *K_V_* up to 10 will be assigned if this is true within a 10 sampling point window. The algorithm also examines the fitting of peak to isotope pattern. Another ad hoc score *K_I_* will be assigned. The summarizing score *S_Cf_* = *ScK_V_K_I_* is reported for each peptide peak candidate. Finally a threshold is used on the summarizing score to differentiate peptide and non-peptide peaks.

#### 2.2.2. Vectorized Peak Detection

Vectorized peak detection [34] is a relatively simple method of identifying peaks in LC/MS data. The aim of the technique is to identify areas in the data either directly as peaks or as an adjunct to other methods of peak detection.

The algorithm has but a single operating rule. For an m/z-retention-time pair to be classified as part of a peak, it must be present in both the MS spectrum of that retention time and the LC data containing that m/z value. Data pairs thus identified can then be further optimized or filtered, if desired.

Hastings, *et al.* reported the method to be significantly more robust when compared with another method, AUTOPSY. As any detected peaks must be present in both the LC and MS dimensions of the data, then, in their requisite places, solvent cluster or column bleed contaminations in the LC data would generally not be detected as peaks, since those contaminations would also need to be present in the MS data. Likewise, chemical or instrumental detector noise in the spectrometry data would not generally be detected as peaks. In effect, this results in an adaptive noise threshold significantly more useful than methods such as global thresholding.

The method is also reported to be suitable for use with other peak detection methods. While specific merging with other methods was not discussed in detail, it would appear that this method would be usable as either a precursor to another method or as a post-processor on candidate peaks already identified in one or the other sets of data. Further, undoubtedly due to its simplicity, it was reported that other considerations such as line shape and isotope distribution could be so incorporated.

#### 2.2.3. MZmine

MZmine [35, 36] is a software package for differential LC/MS analysis. It is a collection of software tools for visualization, peak picking, and statistical analysis of LC/MS datasets. It supports many options in peak picking. MZmine has default threshold values but the user can specify all the parameters. It gives the user three algorithms to select from for finding the mass values, two options for constructing the chromatogram, and seven algorithm options for peak recognition. MZmine is not automated so the user must specify the proper parameters and options for their data. It is written in Java by teams from Japan and Finland.

The peak detection algorithm in MZmine is based on peak shape and intensity in both the LC and MS dimensions. It also uses peak width of the LC dimension to differentiate peptide peaks from noise peaks.

Peak detection in MZmine is performed in a three-step manner. First, mass values are detected within each spectrum (several methods are available, depending on the nature of the data). In the second step, a chromatogram is constructed for each of the mass values which span over a certain time range. Finally, deconvolution algorithms are applied to each chromatogram to recognize the actual chromatographic peaks. It also optionally uses a gap filter to fill gaps in a peak list.

In MZmine the user has the option of smoothing the raw data to remove noise from either the LC or MS spectrum. This step is dependent on the type of data and can be ignored if the data is available as centroids. Smooth filtering options are crop filter, mean filter, Savitzky-Golay filter, and chromatographic median filter.

Next, the algorithm processes MS scans and detects possible mass values where a peptide peak may exist. This process is called the mass detection process. The options available are centroid, exact mass, local maximum, recursive threshold, and wavelet transform. For centroid data every point is considered a MS peak. The user must choose an intensity threshold value to filter out lower intensity peaks. For the exact mass detector, the algorithm searches for the FWHM data points and the user specifies the noise level, mass resolution, and peak model function. The local maximum finds the maximum intensities of the current spectrum and discards peaks that are not above noise level. Noise level is defined by the user. In recursive mass detector the user must input the noise level, minimum m/z peak width, and maximum m/z peak width. This method then looks for local maxima that satisfy the given parameters. The wavelet transform mass detector uses the Mexican Hat wavelet. The user must input the noise level, scale level, and wavelet window size. The MS scan is processed through wavelet transformation and local maxima are detected through the transformed data. The software allows users to select MS peak shape (such as Gaussian or Gaussian plus a base triangle); however, it is not clear how this peak shape information is incorporated in the peak picking process.

In the second step, XICs are obtained based on the mass list generated in the first step. There are two options for building chromatograms, the simple connector and the highest datapoint connector. The simple connector connects the mass peaks along the retention time dimension based on a match score. It requires user input of minimum time span and m/z tolerance window which determine the match score. The highest intensity chromatogram builder is similar except that it uses only the highest intensity as its match score. The exact details of how the match score is calculated is not given.

The last step in the peak detection algorithm is peak recognition in the LC dimension. The seven options for peak recognition are: no recognition, baseline, chromatographic threshold, noise amplitude, standard deviation, Savitzky-Golay, and wavelet transform. The no recognition option makes no further processing and forms a LC peak using all mass spectrum peaks connected in the chromatogram. The baseline peak recognition sets a baseline and cuts off any point below the baseline from the chromatogram. The parameters are minimum peak height, minimum peak duration, and baseline level and the algorithm recognizes a chromatographic peak based on these parameters. The chromatographic threshold peak recognition uses a threshold level to use as a baseline and is very similar to baseline peak recognition. The parameters for threshold peak recognition are minimum peak height, minimum peak duration, and chromatographic threshold level. The noise amplitude peak recognition uses the noise amplitude to set the baseline. The parameters are minimum peak height, minimum peak duration, and amplitude of noise. Standard deviation peak recognition sets the baseline level based on the standard deviation of the signal. The parameters are minimum peak height, minimum peak duration, and standard deviation threshold level. Savitzky-Golay peak recognition uses the Savitzky-Golay polynomial to determine the peaks. It has parameters of minimum peak height and minimum peak duration. The wavelet transform peak recognition uses the Mexican Hat wavelet. It has parameters of minimum peak height, minimum peak duration, and wavelet threshold level. The software allows users to select peak shape (such as Gaussian or Exponentially Modified Gaussian) in peak detection in the LC dimension, however, the documentation on peak shape fitting is missing.

After peak detection each LC/MS run *s* from *s* = 1...*S* in LC dimension is stored in a peak list $P_{s}=\left\{p_{\mathrm{isc}}\right\}$ with *i*=1....*N_s_* and *s*=1....*S* and $c=\left\{\mathrm{mz},\delta \mathrm{mz},\mathrm{rt},\delta \mathrm{rt},\mathrm{height},\mathrm{area}\right\}$ where *N_s_* is the total number of peaks in run *s*, *mz* is the mean m/z value for the data points within the peak, *δmz* is the standard deviation of m/z values within the peak, *rt* is the retention time at the maximum intensity data point, *δrt* is the length of the peak in time, *height* is the height of the peak, and *area* is the area of the peak. The area is calculated by the sum of all intensities of the peak.

### 2.3. Two-Dimensional LC/MS Methods

In this category, peak detection algorithms utilize the 2-D nature of LC/MS and perform processing either in the LC dimension or on the 2-D LC/MS image first. Isotope pattern matching is usually performed as the last step or for reporting purposes only. The claimed advantage is that elution time processing first will reduce the noise level greatly and thus increase the accuracy of isotope pattern matching in the m/z dimension. Note that algorithms such as LCMS-2D still process LC data in a 1-D by 1-D fashion.

#### 2.3.1. LCMS-2D

LCMS-2D [37] processes LC/MS datasets in the LC dimension first and then in the m/z dimension. It claims that it performs 2-D processing in contrast to 1-D processing.

The algorithm differentiates peptide and noise peaks based on LC peak frequency content, LC peak intensity, LC peak width, and the fitness to isotope patterns in the m/z dimension. The algorithm assumes that LC peaks have low frequency content and performs smoothing to remove high frequency components. It also uses a preset threshold on the LC peak intensity to filter out peaks with low intensity. The algorithm assumes that peptide peaks span 5-300 scans and LC peaks narrower or wider are considered as noise. (The algorithm suggests to adjust this range in different LC conditions). The algorithm considers overlapping isotope patterns in the m/z dimension for deisotoping and charge state deconvolution using a variable selection algorithm [38].

The algorithm first performs moving average smoothing in the LC dimension. The moving average window size is chosen to be between 3 and the minimum expected peak width. After LC dimension smoothing, each MS scan is converted to a single scan peak list with a method not described. These single scan peak lists include noise peaks. Next, all single scan peak lists are pooled together to form a super list. The peaks are ordered according to their intensity. Starting from the highest intensity single scan peak, an XIC is constructed within a +/- dm window of the m/z of the single scan peak. Then a preset threshold on SNR (2.5) is applied to the XIC to identify LC peaks. The method for determining noise variance is not provided. After thresholding, the LC peak list is further reduced by eliminating peaks with width outside the preset range (3-500 scans). After LC dimension processing, the algorithm proceeds to m/z dimension processing.

The algorithm first pools LC peaks with similar retention time into clusters. Two LC peaks are considered similar in retention time if the peak apexes are within 5 scans, or if for any peak, more than half of the peak area above the half peak height overlaps with that of another peak. Then for elution time profiles within the cluster, it performs deisotoping and charge state deconvolution using a variable selection procedure [38] in the m/z dimension. It is not clearly described which m/z scans within the cluster of the LC peaks are selected for deisotoping. The variable selection deisotoping/deconvolution algorithm considers the observed MS peaks within an m/z window as the superposition of several isotope patterns with different charge states. Each contributing isotope pattern has an intensity value and is considered as a variable. The minimum number of variables that can best explain the observed MS peak is considered the true solution. This method is good for resolving overlapping isotope patterns, a phenomenon very common in lower resolution LC/MS datasets. The isotope patterns are also calculated using the “averagine” [20, 39].

For clusters that cannot be explained well by overlapping isotope patterns, peak picking is performed in the MS dimension directly. The scan at the elution peak apex is used for this purpose.

This method is reported to perform better than pepList and msInspect when testing on a 16 synthetic peptide mixture.

#### 2.3.2. MapQuand

MapQuand is developed by Harvard Medical School [24]. The algorithm differentiates peptide and noise peaks based on frequency and peak intensity. The algorithm assumes that elution and MS peaks have low frequency and filtering is applied. Filtering options include matched filtering using Gaussian curve, box-car, or Savitzky-Golay in both the elution time and MS dimension. The algorithm applies thresholds on intensity to differentiate peptide and non-peptide peaks. The threshold on intensity is based on the mean or median plus standard deviation of the 2-D LC/MS data map *I* , and it shall be adapted to local noise characteristics. The algorithm assumes that a peptide forms 2-D Gaussian curves supported on the 2-D space spanned by the elution time and m/z. Curve fitting is performed to extract peptide peak parameters such as abundance, retention-time centroid, m/z centroid etc. The peaks are deisotoped by fitting isotope patterns to the observed 2-D data. However, the algorithm does not use peak shape or the fitness to isotope pattern to differentiate peptide and non-peptide peaks.

The algorithm first performs smoothing in both the MS and LC dimensions. The smoothed dataset is called a 2-D map. Using a watershed segmentation algorithm [24], the 2-D image is partitioned into different segments. Each segment either contains one 2-D peak or several overlapping 2-D peaks. The goal of segmentation is to reduce computational complexity. Next, within each 2D segment, an algorithm for finding local maxima is employed. A point is decided to be a local maximum if it is greater than N neighboring data points. The definition of the neighboring data points is subject to user definition. These local maxima are considered as peak candidates. To reduce false positives, an intensity threshold is applied to these peak candidates. The threshold is set as the median plus two or three times the average absolute deviation from the median. The process of determining the median and deviation is not described. Subsequently, in order to report important peak parameters, each local maximum in a segment is fitted with a 2-D Gaussian curve:

$f\left(m,r,A,r_{0},m_{0},\sigma_{m},\sigma_{r}\right)=\frac{A}{2\pi \sigma_{m}\sigma_{r}}$

(2) $$e^{\frac{{\left(r-r_{0}\right)}^{2}}{{2\sigma }_{r}^{2}}}e^{\frac{{\left(m-m_{0}\right)}^{2}}{{2\sigma }_{m}^{2}}}$$

where *A* stands for the peak height, *r*_0_,*m*_0_ are peak centroid in the elution time and m/z dimension, and *σ_r_*, *σ_m_* are the deviation in the two dimensions. Non-linear least-squares regression is used for curve fitting. To address the problem that sometimes LC peaks have heavy tails, there is the choice of fitting peaks with the exponentially modified Gaussian (EMG) curve in the LC dimension.

If the peaks from an isotope cluster overlap with one another in low-resolution MS data, an additional curve fitting step that fits a wide 2-D curve is used based on the following parametric model:

$f\left(m,r,A,r_{0},m_{0},\sigma_{m},\sigma_{r},c,z\right)=A\sum_{i}\frac{B\left(i,c,p\right)}{2\pi \sigma_{m}\sigma_{r}}$

$e^{\frac{{\left(r-r_{0}\right)}^{2}}{{2\sigma }_{r}^{2}}}e^{\frac{{\left(m-m_{0}+1/z\right)}^{2}}{{2\sigma }_{m}^{2}}}$

where *B*(*i,c,p*)=(*ci*)*p^cλi^*(1λ*p*)*^i^* is the predicted isotope pattern or the probability of having *i* *C*^13^s out of a total of *c* carbons when the *C*^13^ abundance is *p* . *z* stands for the charge state. The unique aspect of MapQuand is that it does not use fixed abundance probability of *C*^13^ and the number of carbons while most other isotope pattern matching algorithms chose a fixed value for *p* and *c*.

#### 2.3.3. msInspect

msInspect [40] is an open source suite of algorithms for comprehensive analysis of LC/MS data. It is written in Java by LabKey Software, Fred Hutchinson Cancer Research Center, and the University of Washington in Seattle, WA. This software has modular components for signal processing, time alignment, and normalization algorithms that can be replaced without altering the framework.

Their peak detection algorithm within the software package is primarily based on isotope pattern matching, filtering in LC and MS dimensions, LC peak length profile, and peak intensity, differentiating peptide peaks from non-peptide peaks. Their isotope pattern matching algorithm is based on a Kullback-Leibler (KL) deviance score.

The algorithm assumes that elution and MS peaks have low frequency. Smoothing is performed in both LC and MS dimensions to remove high frequency noise. It also assumes that peptide peaks will sustain over time and LC peaks that last too short are noise peaks. The algorithm assumes that the LC peaks of isotopes maximize and disappear at the same time. In the MS dimension, the observed and expected isotopic distributions are compared using KL deviance score which can be used as measure of confidence for detected peptide peaks.

The algorithm first re-samples the raw LC/MS data to index the image. After re-sampling, the algorithm “conservatively” estimates the background level and uses a intensity threshold based on the estimated noise level to remove noise in both the LC and MS dimensions. The method for estimating noise level is not described. Next, peaks in the m/z dimension are identified using a wavelet additive decomposition [41] and reduced to centroid peak lists.

Subsequently peaks in the LC dimension are smoothed. LC peaks that are sustained over time are considered as peptide candidate peaks. LC peaks that appear to maximize and disappear at the same time are pooled together and are considered as isotopes. Any observed isotopic distributions will be extracted and are stored as *P*_m/z,z_.

${\hat{P}}_{\mathrm{mz}}\left(x\right)=\frac{I\left(\mathrm{mz}+x/z\right)}{\sum_{x=0}^{d-1}I\left(\mathrm{mz}+x/z\right)}$                            

*for* $x\in \left\{1...\left(d-1\right)\right\}$ Where the maximum intensity is denoted by *I*(*mz*) and with eluting isotopes being *I*(*mz + x*) $x\in \left\{1...d-1\right\}$ . The default tolerance window chosen is *d* = 6 resulting in at most 5 isotopes.

The theoretical expected isotopic distribution of a peptide of mass m are stored into *P_m_*. The model for the expected isotope distribution is defined as

$P_{m}\left(x\right)=\frac{1}{K_{d}}\frac{\lambda m}{x!}\exp\left(-\lambda m\right)$

*for x*=0,...,(*d-1*) where $\lambda =\frac{1}{1800}$ is based on its fit to the theoretical isotopic distributions calculated from 539 957 tryptic peptides from the human proteome sequence database. *K_d_* is a normalizing constant for *d* eluting isotopes. To compare the closeness between the observed and the modeled distributions a KL score is used. This score measures discrimination information of the two distributions. It is defined by

(3) $$\mathrm{KL}=\sum_{x}\underset{\mathrm{Weight}}{\underset{︸}{\hat{P}\left(\mathrm{mz}+x/z\right)}}\log\underset{\mathrm{Penalty}}{\underset{︸}{\left(\frac{\hat{P}\left(\mathrm{mz}+x/z\right)}{P_{m}\left(x\right)}\right).}}$$

Isotope peaks with the lowest KL value to expected peptide isotope distribution are removed from the peak cluster until all observed isotopes are assigned. Isotopes that are not assigned are given a charge state of zero. If isotopes are within 10% of each other then preference is given to those with lower m/z value and higher charge state. Quantification uses the highest peak within each peptide. It can also use the maximum intensity, the intensity summed over all elution profiles, and the intensities summed over multiple charge states of the same peptide. The algorithm finally produces a peptide feature file which locates each peptide and gives its charge state(s), time at maximum intensity, signal intensity, KL, number of isotopes identified, and the peptide's first and last scan.

## 3. SIMULATION

The LC/MS dataset used for testing various peak picking criteria was generated using the Proteomics Standard Set (UPS1) from *SIGMA λ ALDRICH^TM^*. The UPS1 set is comprised of one vial of Proteomics Standard and one vial (20 mg) of Proteomics Grade Trypsin. The Proteomics Standard is produced from a mixture of 48 individual human source or human sequence recombinant proteins, each of which has been selected to limit heterogeneous post-translational modifications (PTMs). The total protein content in each vial is 10.6 mg. Each protein has been quantified by amino acid analysis (AAA) prior to formulation.

The UPS1 sample was analyzed using an FTMS mass spectrometer (LTQ-Orbitrap-XL, ThermoFisher, San Jose, CA).

Note that both LC/MS and LC/MS/MS scans were collected in this experiment; however, this does not cause discontinuities in the elution profiles in the LC/MS data because LC/MS and LC/MS/MS data are collected in different sections of the same instrument. Even though the protein content in the UPS1 sample is known, the exact set of peptides after trypsin digestion cannot be theoretically predicted exactly due to complications such as missed cleavages and oxidation. The generated dataset is converted from the manufacturer's proprietary file format to mzXML.

To annotated the LC/MS data, i.e., to assign amino acid sequences to peptides, LC/MS/MS data was searched with the MASCOT protein identification algorithm. LC/MS/MS peaklist is searched in Mascot. Mascot returns a list of probable proteins based on MS/MS spectrum for each peptide. The Mascot search result is then compared to the original protein list. Out of the 283 probable proteins returned by the Mascot search results, 46 out of 48 proteins contained in the original sample are present. We treat the set of observed peptides in LC/MS/MS scans associated with these 46 proteins as the set of “true peptides” denoted as *L_peptide_* with size *N_p_* that is contained in the trypsin digested sample. Note that this list of 800 peptides cannot be the complete set of peptides contained in the sample; however, it is a very close approximation which can be used to compare the performance of various peak picking algorithms.

We tested the effect of various peak detection methods based on the peak list generated by the peak detection algorithms in msInspect, MZmine as well as that generated by the algorithm described in VIPER software which we implemented. The purpose of the test is to illustrate the effect of using various peak picking criteria rather than comparing the performance of software packages. Since many software packages allow the selection of different peak picking criteria, direct comparison of their performance is impossible.

In this section, we evaluate the performance of peak picking criteria based on isotope pattern matching, intensity, signal-to-noise ratio and LC peak shape matching. A fair way of conducting the comparison these methods is to compare their ROC curve, i.e., plot the false positive rate vs. true positive rate as the threshold on parameters varies. Suppose the list of all peaks contained in an LC/MS data set is *D* with *N_d_* peaks. Each item in the list is indexed by its mass and then followed by parameters such as isotope pattern matching score. Then a threshold on one of the parameters such as isotope matching score can be applied. Peaks pass the threshold will be treated as peaks detected. Detected peaks are then partitioned to a set of *N_t_* “true” peaks and *N_f_* “false” peaks by comparing the detected peaks with the set of “true peptides”. The true positive rate is estimated as *N_t_/N_p_*, which indicates the probability of detecting a true peak. The false positive rate is estimated as *N_f_*/(*N_d_*λ*N_p_*), which indicates the probability of false peaks being detected as true peaks. The performance of a peak picking algorithm is better when its true detection rate is higher at a given false detection rate. This is reflected as the area under the curve; the larger it is, the better the performance is.

We elected to use the peak list generated by msInspect, MZmine and the peak detection algorithm described in VIPER for testing. msInspect was selected because by setting up all thresholds to their minimum or maximum values the algorithm can report all peptide and noise peaks existing in the data. msInspect reports the intensity, LC peak duration, isotope matching score (KL distance), background noise etc. These parameters allow us to perform peak detection by applying thresholds on specific parameters and evaluate the effectiveness. For example, if we want to evaluate the performance of peak detection algorithms based on isotope matching, we can apply a threshold to KL distances of the list of candidate peptides reported by msInspect. The MZmine peptide list is used for evaluating the effect of shape filtering in the LC domain. We implemented the peak picking algorithm described in VIPER which is basically a 1-D isotope matching algorithm based on the minimum mean square error criterion. The purpose was to corroborate the performance curves derived using msInspect. Good correlation between the two algorithms when they use the same peak picking parameter was found, which provides confidence of our evaluation methods.

### 3.1. Performance of Peak Detection based on Isotope Pattern Matching

Many software packages such as msInspect and VIPER provide the option of detecting peaks based on isotope matching. Different isotope matching metrics such as KL distance or mean square error can be used. msInspect performs LC dimension filtering while the peak detection algorithm in VIPER does not. The ROC curves are shown in Fig. (**9**). From this we can see that using KL distance or mean square distance provides similar performance. The performance gain of msInspect in the lower detection region could be attributed either to KL distance as a better criteria or because of the LC dimension filtering performed in msInspect.

### 3.2. Performance of Peak Detection Based on Various Pick Picking Criteria

Besides isotope pattern matching, most commercial and open source software packages such as msInspect offer a variety of peak picking criteria. Here we examine the performance of peak picking by applying thresholds on peak intensity, scan count (LC peak length), KL distance, Total peak intensity, and the signal-to-noise ratio (SNR) based on the msInspect peptide list. The SNR is calculated as the squared ratio between peak intensity and the background. msInspect reports a background level of zero for some peaks in the peaklist. In such cases, we treat these peaks as noise peaks. The results are shown in Fig. (**10**), and we can see that the best performance is achieved by using intensity. The worst performance is based on SNR. This graph suggests an inverse growth of SNR as peak intensity grows.

### 3.3. Combining Peak Detection Criteria

Next, we examine the effect of combining two criteria. The effect of applying thresholds in scan count (LC peak length) and then on intensity is shown in Fig. (**11**).

From Fig. (**11**), it can be seen that applying threshold on scan count first does not improve the ROC curve. On the other hand, applying threshold on intensity first does provide some improvement on the ROC curve at a cost of the limited detection rate. This shows that peptide and noise peak have a more pronounced difference in peak intensity then in LC peak length. Scan count is correlated with peak intensity and is largely a redundant feature. Note however, using intensity as the threshold will be less effective if the peptide abundance approaches the noise level.

We have also investigated the effect of combining KL distance and intensity for peak detection. The result is very similar to the combination of scan count and intensity. It seems that peak picking based on isotope matching does not improve the overall accuracy and thresholding on intensity alone is good enough for detecting peaks.

### 3.4. Peak Detection with LC Peak Shape Filtering

The software packages MZmine and MapQuand provide the capability of peak picking based on LC peak shape. LC peak shape that does not conform to a pre-defined template is discarded as noise. It is anticipated that the performance will be very bad for the LC/MS dataset that we have since it has many irregular LC peak shapes. We utilized the peak list generated by the MZmine software. The software allows the option of using an Extended Gaussian template for detecting LC peaks. The peak list generated by MZmineine is not de-isotoped. The ROC based on thresholding on intensity is shown in Fig. (**12**). We can see that the performance is much worse than that of msInspect which did not perform LC domain peak filtering based on peak shape.

## 4. DISCUSSION AND FUTURE RESEARCH

We have reviewed popular LC/MS peak picking algorithms in the literature. They are categorized based on their main processing methods. The impact of different peak picking methods is examined by comparing results for a 48 protein mixture LC/MS dataset. The simulations show that intensity seems to be the most effective criteria for peak detection. Other criteria such as the length of LC peak, LC peak shape, and the isotope matching score do not improve the overall accuracy with the given dataset. This is somewhat surprising since we anticipated that more than one criteria should improve the accuracy if they are combined appropriately.

The obvious deficiencies of current peak picking algorithms mainly lie in two aspects. One is that there lacks an accurate and complete model for peptide and noise peaks. All current algorithms are developed based on partial models of the peptides. For example, isotope matching algorithms only partially model peptide peaks at each charge state. As a result, peak detection is conducted for each charge state separately. Thus the low abundance charge state peaks of a peptide may not be correctly linked to the rest of the charge states and are often wrongly detected as another peptide with a different mass. Models adopted by some of the current algorithms are also inaccurate. For example, the MapQuand algorithm assumes a two dimensional correlated Gaussian model for peptide peaks. However, we know that the LC elution process is conducted first and independently from the MS process, and the resulting peaks are not correlated in the two dimensions. Thus the model is unnecessarily complicated. The assumption of a Gaussian shape for LC peaks is also inaccurate under many experimental conditions. The study of the noise model is also lacking. Results of noise model study often conflicts one another [23].

Another aspect is the ad hoc nature of current peak picking algorithms, which leads to poor performance. For example, the isotope pattern of a peptide is registered in multiple MS scans during the eluting period of the peptide. Information from different MS scans can be combined together for isotope pattern estimation. However, none of the current algorithms perform isotope pattern matching based on multiple MS scans.

We anticipate that significant performance improvement can be achieved by constructing accurate and complete models as well as performing near-optimal processing based on the models.

## Figures and Tables

**Fig. (3):**
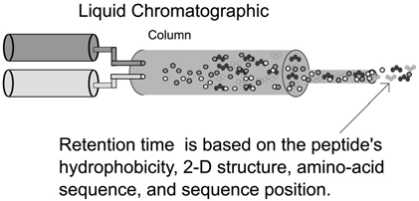
The LC elution column.

**Fig. (4):**
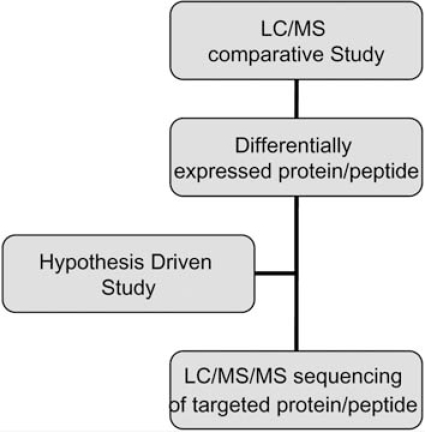
Example of LC/MS Work Flow.

**Fig. (5):**
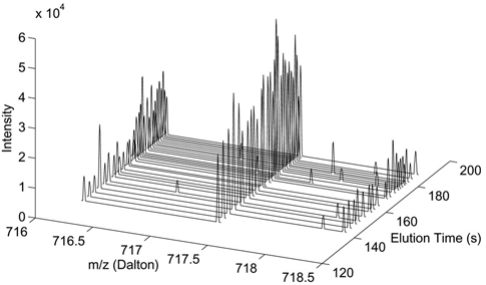
Example of LC/MS dataset.

**Fig. (6):**
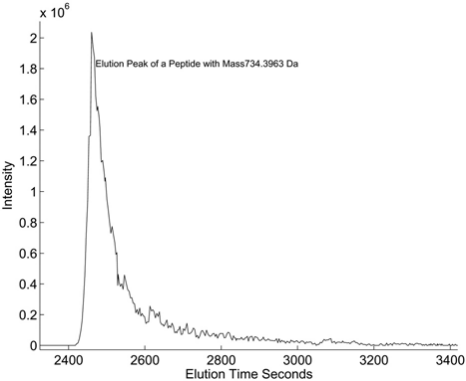
Example of LC/MS elution time profile.

**Fig. (7):**
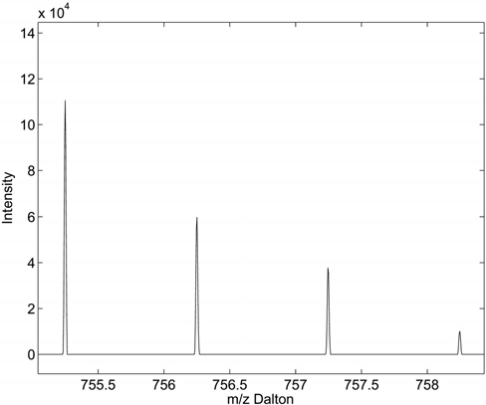
Example of an observed isotope pattern in a MS scan.

**Fig. (8):**
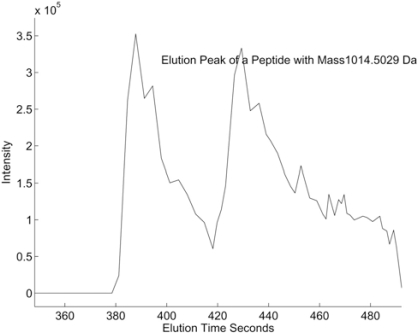
Example of LC/MS elution time profile with 2 peaks.

**Fig. (9):**
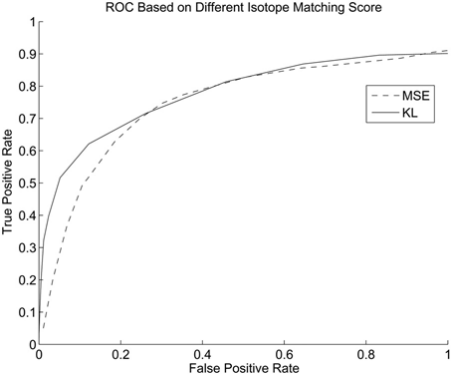
ROC curve based on KL distance and mean square error (MSE).

**Fig. (10):**
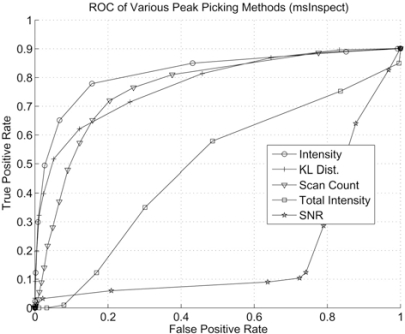
ROC curve with thresholds on different peak picking criteria.

**Fig. (11):**
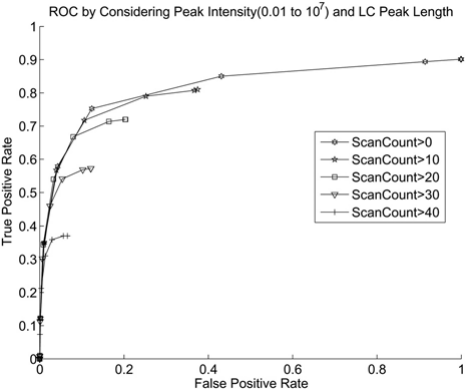
ROC curve with thresholds on scan count and then on intensity.

**Fig. (12):**
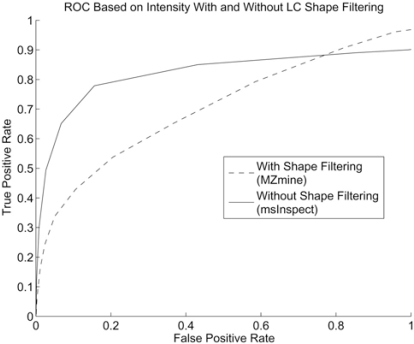
ROC with and without LC peak shape filtering.

## References

[1] Tyers M, Mann M (2003). From genomics to proteomics. Nature.

[2] Aebersold R, Mann M (2003). Mass spectrometry-based proteomics. Nature.

[3] Hanash S (2003). Disease proteomics. Nature.

[4] Boguski MS, McIntosh MW (2003). Biomedical informatics for proteomics. Nature.

[5] Gross ML, Rempel DL (1984). Fourier transform mass spectrometry. Science.

[6] Chernushevich IV, Loboda AV, Thomson BA (2001). An introduction to quadrupole-time-of-flight mass spectrometry. J. Mass Spectrom.

[7] Hop C, Bakhtiar R (1997). An Introduction to Electrospray Ionization and Matrix-Assisted Laser Desorption/Ionization Mass Spectrometry: Essential Tools in a Modern Biotechnology Environment. Biospectroscopy.

[8] Batoy S, Akhmetova E, Miladinovic S, Smeal J, Wilkins CL (2008). Developments in MALDI Mass Spectrometry: The Quest for the Perfect Matrix. Appl. Spectrosc. Rev.

[9] Bruins AP, Covey TR, Henion JD (1987). Ion spray interface for combined liquid chromatography/atmospheric pressure ionization mass spectrometry. Anal. Chem.

[10] Arpino P (1992). Combined liquid chromatography mass spectrometry. Part 111. Applications of thermospray. Mass Spectrom. Rev.

[11] Roy SM, Anderle M, Lin H, Becker CH (2004). Differential expression profiling of serum proteins and metabolites for biomarker discovery. Int. J. Mass Spectrom.

[12] Regnier F, Huang G (1996). Future potential of targeted component analysis by multidimensional liquid chromatography-mass spectrometry. J. Chromatogr. A.

[13] Wu SL, Amato H, Biringer R, Choudhary G, Shieh P, Hancock WS (2002). Targeted proteomics of low-level proteins in human plasma by LC/MSn: using human growth hormone as a model system. J. Proteome Res.

[14] Kislinger T, Cox B, Kannan A, Chung C, Hu P, Ignatchenko A, Scott MS, Gramolini AO, Morris Q, Hallett MT, Rossant J, Hughes TR, Frey B, Emili A (2006). Global Survey of Organ and Organelle Protein Expression in Mouse: Combined Proteomic and Transcriptomic Profiling. Cell.

[15] Adkins JN, Varnum SM, Auberry KJ, Moore RJ, Angell NH, Smith RD, Springer DL, Pounds JG (2002). Toward a Human Blood Serum Proteome Analysis By Multidimensional Separation Coupled With Mass Spectrometry* S. Mol. Cell. Proteom.

[16] Liu H, Sadygov RG, Yates III JR (1999). A Model for Random Sampling and Estimation of Relative Protein Abundance in Shotgun Proteomics. Nat. Biotechnol.

[17] Valkenborg D, Assam P, Thomas G, Krols L, Kas K, Burzykowski T (2007). Using a Poisson approximation to predict the isotopic distribution of sulphur-containing peptides in a peptide-centric proteomic approach. Rapid Commun. Mass Spectrom.

[18] Bayne CK, Smith DH (1984). A new method for estimating isotopic ratios from pulse-counting mass spectrometric data. Int. J. Mass Spectrom. Ion Process.

[19] Senko MW, Beu SC, McLafferty FW (1995). Determination of monoisotopic masses and ion populations for large biomolecules from resolved isotopic distributions. J. Am. Soc. Mass Spectrom.

[20] Rockwood AL, Van Ordent SL, Smith RD (1996). Ultrahigh Resolution Isotope Distribution Calculations. Rapid Commun. Mass Spectrom.

[21] Horn DM, Zubarev RA, McLafferty FW (2000). Automated reduction and interpretation of high resolution electrospray mass spectra of large molecules. J. Am. Soc. Mass Spectrom.

[22] Coombes KR, Koomen JM, Baggerly KA, Morris JS, Kobayashi R (2005). Understanding the characteristics of mass spectrometry data through the use of simulation. Cancer Inform.

[23] Du P, Stolovitzky G, Horvatovich P, Bischoff R, Lim J, Suits F (2008). A noise model for mass spectrometry based proteomics. Bioinformatics.

[24] Leptos KC, Sarracino DA, Jaffe JD, Krastins B, Church GM (2006). MapQuant: Open-source software for large-scale protein quantification. Proteomics.

[25] Andreev VP, Rejtar T, Chen HS, Moskovets EV, Ivanov AR, Karger BL (2003). A Universal Denoising and Peak Picking Algorithm for LC-MS Based on Matched Filtration in the Chromatographic Time Domain. Anal. Chem.

[26] Gras R, Mueller M, Gasteiger E, Gay S, Binz PA, Bienvenut W, Hoogland C, Sanchez JC, Bairoch A, Hochstrasser DF (1999). Improving protein identification from peptide mass fingerprinting through a parameterized multi-level scoring algorithm and an optimized peak detection. Electrophoresis.

[27] Zhang Z, Guan S, Marshall AG (1997). Enhancement of the effective resolution of mass spectra of high-mass biomolecules by maximum entropy-based deconvolution to eliminate the isotopic natural abundance distribution. J. Am. Soc. Mass Spectrom.

[28] Monroe ME, Tolic N, Jaitly N, Shaw JL, Adkins JN, Smith RD (2007). VIPER: an advanced software package to support high-throughput LC-MS peptide identification. Bioinformatics.

[29] Mueller LN, Rinner O, Schmidt A, Letarte S, Bodenmiller B, Brusniak MY, Vitek O, Aebersold R, Muller M (2007). SuperHim-a novel tool for high resolution LC-MS-based peptide/protein profiling. Proteomics.

[30] Sturm M, Bertsch A, Groepl C, Hildebrandt A, Hussong R, Lange E, Pfeifer N, Schulz-Trieglaff O, Zerck A, Reinert K, Kohlbacher O (2008). OpenMS-An open-source software framework for mass spectrometry. BMC Bioinformatics.

[31] Li X, Yi EC, Kemp CJ, Zhang H, Aebersold R (2005). A Software Suite for the Generation and Comparison of Peptide Arrays from Sets of Data Collected by Liquid Chromatography-Mass Spectrometry* S. Mol. Cell. Proteom.

[32] Coifman RR, Donoho DL (1994). Translation invariant de-noising. Wavelets and Statistics. Springer Lecures Notes in Statistics.

[33] Li X, Zhang H, Ranish JA, Aebersold R (2003). Automated Statistical Analysis of Protein Abundance Ratios from Data Generated by Stable-Isotope Dilution and Tandem Mass Spectrometry. Anal. Chem.

[34] Hastings CA, Norton SM, Roy S (2002). New algorithms for processing and peak detection in liquid chromatography/mass spectrometry data. Rapid Comm. Mass Spectrom.

[35] Katajamaa M, Oresic M (2005). Processing methods for differential analysis of LC/MS profile data. BMC Bioinformatics.

[36] Katajamaa M, Miettinen J, Oresic M (2006). MZmine: toolbox for processing and visualization of mass spectrometry based molecular profile data. Bioinformatics.

[37] Du P, Sudha R, Prystowsky MB, Angeletti RH (2007). Data reduction of isotope-resolved LC-MS spectra. Bioinformatics.

[38] Du P, Angeletti RH (2006). Automatic deconvolution of isotope-resolved mass spectra using variable selection and quantized peptide mass distribution. Anal. Chem.

[39] Hochstrasser DF, Appel RD (1999). Modeling peptide mass fingerprinting data using the atomic composition of peptides. Electrophoresis.

[40] Bellew M, Coram M, Fitzgibbon M, Igra M, Randolph T, Wang P, May D, Eng J, Fang R, Lin CW, Chen J, Goodlett D, Whiteaker J, Paulovich A, McIntosh M (2006). A suite of algorithms for the comprehensive analysis of complex protein mixtures using high-resolution LC-MS. Bioinformatics.

[41] Yu W, Wu B, Lin N, Stone K, Williams K, Zhao H (2006). Detecting and aligning peaks in mass spectrometry data with applications to MALDI. Comput. Biol. Chem.

